# Effectiveness of a web platform on university students’ motivation to quit smoking

**DOI:** 10.1590/1518-8345.3731.3318

**Published:** 2020-07-01

**Authors:** Alba María Romero-López, Silvia Portero-de-la-Cruz, Manuel Vaquero-Abellán

**Affiliations:** 1 Universidad de Córdoba, Facultad de Medicina y Enfermería, Departamento de Enfermería, Farmacología y Fisioterapia, Córdoba, Andalucía, Spain.

**Keywords:** Tobacco Use Disorder, Nicotine, Information Technology, Motivation, Tobacco Use Cessation, Students, Health Occupations, Tabagismo, Nicotina, Tecnologia da Informação, Motivação, Abandono do Uso de Tabaco, Estudantes de Ciências da Saúde, Tabaquismo, Nicotina, Tecnología de la Información, Motivación, Cese del Uso de Tabaco, Estudiantes del Área de la Salud

## Abstract

**Objective:**

to know the dependence on nicotine and the motivation to quit smoking in Nursing and Physiotherapy students of a university in the South of Spain, and to evaluate the impact of an intervention based on the use of information technologies on the motivation to quit smoking.

**Method:**

a pilot study in two phases: the first being cross-sectional and the second, a before-and-after intervention. The motivation to quit smoking was assessed by means of the Richmond questionnaire, and the dependence on nicotine through the Fagerström questionnaire; additionally, an intervention was performed based on the use of a web platform to increase motivation to quit smoking. Descriptive and inferential statistics were applied.

**Results:**

the prevalence in the use of tobacco was 4.33% (n=29). 3.45% of the participants had a high level of dependence; and 6.90%, a high level of motivation. The level of motivation did not change after the intervention (p=0.10).

**Conclusion:**

most of the students have low levels of motivation to quit smoking and of physical dependence to nicotine. The level of motivation to quit smoking does not change after performing the intervention.

## Abstract (pt)

**Objetivo:**

conhecer a dependência da nicotina e a motivação para parar de fumar em estudantes de Enfermagem e Fisioterapia de uma universidade no sul da Espanha e avaliar o impacto de uma intervenção baseada no uso de tecnologias da informação na motivação para parar de fumar.

**Método:**

estudo piloto em duas fases: a primeira transversal e a segunda de intervenção antes-depois. A motivação para parar de fumar foi avaliada usando o questionário Richmond, a dependência da nicotina através do questionário de Fagerström e uma intervenção baseada no uso de uma plataforma online foi realizada para aumentar a motivação para parar de fumar. Estatística descritiva e inferencial foram aplicadas.

**Resultados:**

a prevalência de consumo de tabaco foi de 4,33% (n=29). 3,45% dos participantes tinham alta dependência e 6,90%, alta motivação. O nível de motivação não foi alterado após a intervenção (p=0,10).

**Conclusão:**

a maioria dos estudantes tem baixo nível de motivação para parar de fumar e dependência física à nicotina. O nível de motivação para parar de fumar não é diferente após a realização da intervenção.

## Abstract (es)

**Objetivo:**

conocer la dependencia a la nicotina y la motivación para el cese tabáquico en estudiantes de Enfermería y Fisioterapia de una universidad del sur de España y evaluar el efecto de una intervención basada en el uso de tecnologías de la información en la motivación para el cese tabáquico.

**Método:**

estudio piloto de dos fases: la primera transversal y la segunda de intervención antes-después. Se valoró la motivación para dejar de fumar mediante el cuestionario Richmond, la dependencia a la nicotina a través del cuestionario Fagerström, y se llevó a cabo una intervención basada en el uso de una plataforma *web* para incrementar la motivación del cese tabáquico. Se aplicó estadística descriptiva e inferencial.

**Resultados:**

la prevalencia de consumo de tabaco fue del 4.33% (n=29). El 3.45% de los participantes presentó alta dependencia, y el 6.90%, alta motivación. El nivel de motivación no se vio alterado tras la intervención (p=0.10).

**Conclusión:**

la mayor parte de los estudiantes tiene un nivel bajo de motivación para dejar de fumar y de dependencia física a la nicotina. El nivel de motivación para el cese tabáquico no es diferente tras realizar la intervención.

## Introduction

The World Health Organization considers smoking as an epidemics with important repercussions on public health and as responsible for over 7,000,000 deaths *per* year, of which 890,000 are due to exposure to the smoke from other people’s tobacco^(^ 1 ^)^.

In Europe, although the use of tobacco in young adults has diminished with time, it is estimated that approximately 10.57%of the individuals over 15 years old smoke daily^(^ 2 ^)^. In Spain, 26% of the individuals between 18 and 24 years old studies in universities^(^ 3 ^-^ 4 ^)^. In Spanish university students, smoking is especially worrisome due to the high prevalence of tobacco use (29.70%)^(^ 5 ^)^. University students consider that tobacco contributes to relaxation and concentration in periods of higher intellectual demand like exams, which leads to a higher consumption of cigarettes per day and to a reason to continue smoking^(^ 6 ^)^. Additionally, living in a university dormitory or in an apartment with other students, being male, and being in the higher courses are also factors associated to a higher consumption in this collective, as well as to a lower level of motivation to quit the habit^(^ 7 ^-^ 8 ^)^.

Currently, it is little probable that the young adults resort to traditional therapies to quit smoking^(^ 9 ^)^. For that reason, innovative interventions which contribute to quitting the smoking habit are essential to reach and involve this population. In this sense, the use of Information and Communication Technologies (ICTs) in the management and prevention of diseases is increasing^(^ 10 ^)^. Numerous studies have signaled that those based on the web and on mobile phones have a positive effect on the motivation to quit smoking and on quitting the habit in university students^(^ 10 ^-^ 12 ^)^. Despite that, only 6.1% of the Spanish population knows about them and about the benefits they bring to people’s health^(^ 13 ^)^.

Conducting this study becomes necessary considering that university students constitute one of groups especially prone to adopting risk behaviors like smoking^(^ 10 ^)^, and that the Health Sciences students will have, as future professionals, an important role in combating smoking by educating the population, supporting anti-tobacco policies, and influencing on the national and global efforts to control tobacco^(^ 14 ^)^.

The objectives of the present study were the following: to know the dependence on nicotine and the motivation to quit smoking in Nursing and Physiotherapy students of a university in the South of Spain, and to evaluate the impact of an intervention based on the use of ICTs in the motivation to quit smoking.

## Method

A two-phase pilot study was conducted: the first phase presented a cross-sectional design to know the dependence on nicotine and the motivation to quit smoking, and the second one had a before-and-after intervention design with the purpose of assessing the impact of an intervention based on the use of information technologies in the motivation to quit smoking. The first phase was carried in January 2019 and the second, in February and March 2019.

The study subjects were Nursing and Physiotherapy students of a university in the South of Spain who were regular smokers, enrolled in any course of the 2018/2019 academic year. As exclusion criteria, it was decided to exclude those students who were immersed in mobility programs, both national and international ones, as well as those who did not understand Spanish.

The students were selected by means of convenience sampling. The study was approved by the Research Ethics Committee of Córdoba (Minutes No. 283, reference 4135).

For the first stage, a dossier made up by the following was designed: (i) an informative letter, in which the voluntary and anonymous nature of participating in the study was highlighted, (ii) an explicit collaboration request in which the students consented to their participation, (iii) an original and specific form which collected sociodemographic and academic variables, (iv) the Fagerström questionnaire^(^ 15 ^-^ 16 ^)^, and (v) the Richmond questionnaire^(^ 17 ^)^, as well as (vi) a sheet in which the web address to which they should navigate to perform the intervention was provided.

The sociodemographic and academic variables collected were the following: gender (male, female), age (years old), course (first, second, third, fourth), degree (Nursing, Physiotherapy), co-living condition during the academic year (with parents, alone in an apartment, with roommates in an apartment, university dormitory), reason for smoking (internal reason: releasing stress/experimenting pleasure/curiosity; external reason: social pressure). The following variables were dichotomized in order to homogenize the sampling size of the subgroups: course (first - third, second - fourth), co-living condition (with parents, outside the family nucleus).

The dependence on nicotine was assessed by means of the Fagerström questionnaire^(^ 15 ^-^ 16 ^)^. This is a self-administered questionnaire made up of 6 items. 4 of the items present dichotomic answers: the difficulty of not smoking in forbidden places (yes, no); if the person smokes more frequently during the first hours of the day (yes, no); if the person smokes although he/she has to stay in bed most of the day (yes, no); and which is the cigarette that most bothers him/her not to smoke (the first in the morning, any other). The other 2 remaining items are classified in a Likert scale from 0 to 3 points. Such items are the following: the time elapsed from the moment he/she wakes up and when he/she smokes (5 minutes, between 6 and 30 minutes, between 31 and 60 minutes, more than 60 minutes); how many cigarettes does the person smoke (<10, 11-20, 21-30, >31). The total score is obtained by summing up the items, and it ranges from 0 to 10 points. Scores lower than or equal to 4 points are considered as low dependence to nicotine, between 5 and 7 points as moderate dependence, and between 8 and 10 points as high dependence.

The motivation to quit smoking was measured by means of the Richmond questionnaire^(^ 17 ^)^. This is a hetero-administered questionnaire made up by 4 items. One of the items has a score from 0 to 1 point: Would you like to quit smoking if you could do it easily? (yes, no). The remaining items have scores from 0 to 3 points: From 0 to 3, state your will to quit it. (none, little, pretty much, a lot); Will you try to quit smoking in the next two weeks? (no, doubtfully, probably, yes); Do you think you will be an ex-smoker in six months’ time? (no, doubtfully, probably, yes). The questionnaire total score is obtained by summing up the items and ranges from 0 to 10 points, with the following assessment: from 0 to 5 points, none or little motivation; from 6 to 7 points, moderate motivation with a need for help; and from 8 to 10, high motivation to quit smoking. This variable was dichotomized in order to homogenize the sampling size of the subgroups, as follows: moderate-low motivation (0 - 7 points) and high motivation (8 - 10 points).

Prior to data collection, contacts were made with the teachers responsible for the subjects involved so as to minimize the interferences in the correct development of the teaching methodology. The planned place, dates, and times to proceed with the handing out of the dossiers to the participants were agreed with the teachers. Such dossiers were handed out to the students in class hours and returned directly to the researchers face-to-face during the class. The students who used tobacco and who accepted to participate in the second phase of the study (before-and-after intervention design) had to write in the questionnaire the last three digits and the letter from their National Identity Card so that the second phase could be carried out and that the participants’ confidentiality and anonymity could be guaranteed.

All of the enrolled students in Nursing and Physiotherapy were surveyed in this phase (670 students), of which 29 used tobacco and agreed to participate in the next phase.

In the second phase, the participants had to visit the web address provided, which was self-made. The content of the web was based on the information provided by the Centers for Disease Control and Prevention^(^ 18 ^)^. The theoretical model used to perform the intervention was the adaptation of the Transtheoretical Model of Change and Behavior^(^ 19 ^)^. This model divides people with health problems into phases according to their level of motivation for change (that is: pre-contemplation, contemplation, preparation, action, and maintenance). According to this, the content of the intervention was designed to increase the smokers’ level of motivation and the probability of an attempt to quit smoking (pre-contemplation/contemplation phase). The intervention consisted in raising the students’ awareness during four weeks on the importance of quitting tobacco use through reading and visualization on the web diverse information on smoking and its health risks, videos in which ex-smokers narrated their life experiences, as well as interaction by means of a space where people could comment and tell their experiences with tobacco. The information contained in the web was updated once a week. During this period of time, the researchers made serialized records of the participants’ activity in the web. The time they were connected (minutes) was counted, as well as if they commented or answered something in the open discussion forum (yes, no).

Two weeks after the end of the awareness-rising phase, they were administered the Richmond questionnaire^(^ 17 ^)^ of motivation to quit smoking through the web, with prior notice of the exact day in which they had to enter to complete it.

Of the 29 students who used tobacco and who accepted to participate in the second phase, 9 continued until the end.

The qualitative variables were expressed as absolute frequencies and percentages, and the quantitative variables as mean values and typical deviations. Data normality was verified using the Shapiro-Wilk test. For comparing mean values between two groups, the Mann-Whitney’s U statistical test was used. The comparison of mean values among more than two groups was performed using the Kruskal-Wallis test. The relation among the qualitative variables was established by the chi-square test. The correlation among variables was carried out by means of the Spearman test. The effectiveness of the intervention was assessed by means of the T-Student test for paired data. In all the statistical tests, p values below 0.05 were considered as significant. The statistical analysis was performed using the G-Stat program, version 2.

## Results

Of the total of students enrolled in the Nursing and Physiotherapy courses (670 students: 496 in Nursing and 174 in Physiotherapy), 29 were smokers, which represented a 4.33% prevalence of tobacco use. Table 1 shows the descriptive data of the studied sample. 65.52% of the students were women, with a mean age of 21.89 (6.38) years old. 68.97% were Nursing students, most of them in second year (41.38%). In relation to their co-living situation, 41.38% lived with their parents. As regards the reason for which they started smoking, 68.97% said it was due to internal reasons (like releasing stress), with up to 86.21% having a low dependence on nicotine. Finally, almost half of the sample (48.28%) stated having a low level of motivation to quit smoking.

**Table 1:** Descriptive, sociodemographic, academic variables. Córdoba, Andalusia, Spain, 2019

Qualitative variables	Frequency	Percentage
Gender Male Female	10 19	34.48 65.52
Course Nursing Physiotherapy	20 9	68.97 31.03
Year First Second Third Fourth	10 12 4 3	34.48 41.38 13.79 10.34
Co-living Parents Shared apartment Alone or with partner in an apartment Residence	12 12 3 2	41.38 41.38 10.34 6.90
Reason for smoking External Internal	9 20	31.03 68.97
Dependence (Fagerström) Low dependence Moderate dependence High dependence	25 3 1	86.21 10.34 3.45
Motivation to quit smoking (Richmond) Low motivation Doubtful motivation Moderate motivation High motivation	14 10 3 2	48.28 34.48 10.34 6.90
**Quantitative variable**	**Mean**	**Typical deviation**
Age (years old)	21.89	6.38

No significant relation was found between dependence on nicotine and gender (p=0.26), course (p=0.64), year (p=0.76), co-living condition (p=0.80), reason to quit smoking (p=0.05), age (p=0.38), or motivation to quit smoking (p=0.61) (Table 2).

**Table 2:** Relation between the dependence on nicotine and the sociodemographic and academic variables. Córdoba, Andalusia, Spain, 2019

Qualitative variables	Level of nicotine dependence (points) (n=29)	
	Mean (Typical deviation)	p value
Gender Male Female	2.80 (2.62) 1.63 (1.80)	0.26
Course Nursing Physiotherapy	2.20 (2.35) 1.67 (1.66)	0.64
Reason External Internal	3.22 (2.54) 1.50 (1.76)	0.05
Co-living With parents Outside the family nucleus	2.33 (2.71) 1.82 (1.70)	0.80
Richmond Low motivation High motivation	1.96 (2.14) 2.40 (2.41)	0.61
Year First Second Third Fourth	2.10 (1.73) 1.92 (2.68) 2.50 (2.08) 1.67 (2.08)	0.76
**Quantitative variable**	**Rho**	**p value**
Age	0.17	0.38

No relation was either found between motivation to quit smoking and gender (p=0.18), course (p=0.56), year (p=0.71), reason to quit smoking (p=0.12), or age (p=0.20) (Table 3).

**Table 3:** Relation between the level of motivation to gradually quit smoking measured with the Richmond questionnaire and the sociodemographic and academic variables. Córdoba, Andalusia, Spain, 2019

Qualitative variables	Level of the motivation to quit smoking (points) (n=29)				
	Low motivation		High motivation		p value
	Frequency	Percentage	Frequency	Percentage	
Gender Male Female	7 17	24.14 58.62	3 2	10.34 6.90	0.18
Course Nursing Physiotherapy	16 8	55.17 27.59	4 1	13.79 3.45	0.56
Reason External Internal	6 18	20.69 62.07	3 2	10.34 6.90	0.12
Co-living With parents Outside the family nucleus	9 15	31.03 51.72	3 2	10.34 6.90	0.35
Year First-Third Second-Fourth	10 14	34.48 48.28	4 1	13.79 3.45	0.12
**Quantitative variables**	**Mean** **(Typical deviation)**		**Mean** **(Typical deviation)**		**p value**
Age (years old)	20.92 (5.35)		26.60 (9.32)		0.20

Of the 29 participants who used tobacco, 48.28% (n=14) started the intervention on the web page provided. Likewise, of the students who started the intervention, those who completed it, including the post-intervention questionnaire, accounted for 64.29% (n=9).

The level of motivation quit smoking before the intervention [3.56(1.81) points] was not significantly modified after the intervention [4.44(2.07) points] (p=0.10). Apart from that, no relation was found between the time spent on the web and the level of motivation to quit smoking (rho=0.26; p=0.50).

## Discussion

In this study, a low prevalence of the smoking habit was obtained in the Nursing and Physiotherapy students, as well a low dependence on nicotine, and a low level of motivation to quit smoking. Additionally, the level of motivation to quit smoking was not significantly reduced after the intervention.

The prevalence of tobacco use found (4.33%) is lower than the one signaled both at the national (26.70%)^(^ 20 ^)^ and international (8.50%)^(^ 21 ^)^ levels. Additionally, most of the students had a low physical dependence on nicotine. This result is similar to that of another study conducted in a population of similar characteristics^(^ 22 ^)^, and almost doubles the one found in another research^(^ 23 ^)^ conducted with university students from Jordan. The sociocultural differences and those related to the climate or to the economy might explain this variation. Likewise, the motivation to quit smoking was low, coinciding with the results in university populations^(^ 24 ^)^. Despite the fact that research on this aspect in university students is limited due to the differences in the patterns and history of consumption and to their self-identification as social smokers^(^ 25 ^)^, the low level of motivation can be explained by the young age of the students, who feel less vulnerable and show no perception of risk, thus not having any plans to quit^(^ 24 ^)^.

In this study, with a predominance of women, no relation was found between gender, physical dependence to nicotine, and motivation to quit smoking. The evidence found identifies that men have a higher prevalence of the smoking habit than women^(^ 21 ^)^. Nevertheless, according to the Spanish National Statistics Institute, in 2018 an increase was reported of the prevalence of tobacco use in the male gender, both in the European population and in the Spanish one^(^ 26 ^)^. On the other hand, the mean age of the study participants is in accordance with the one signaled in other studies conducted with university students^(^ 20 ^,^ 27 ^)^.

As regards the co-living condition, most of the participants lived outside the family nucleus. During their higher studies, many students begin to live in shared apartments or in university dormitories^(^ 28 ^)^. Thus, their roommates have a direct influence on their immediate behavior, encouraging the use of tobacco and reducing the level of motivation to quit the habit^(^ 6 ^-^ 7 ^)^. Despite that, no relation was found in this study between the co-living condition, dependence on nicotine, and motivation to quit smoking.

Whereas most of the participants asserted to have started smoking due to internal reasons, in men tobacco use supposes an authority role in relation to their equals, or a passage to maturity; and women are initiated into smoking because of curiosity or due to social influence. However, in both cases, tobacco use relaxes them and increases their self-confidence^(^ 4 ^)^.

Although no difference was found between the motivation levels to quit smoking before and after the intervention, the efficacy of the interventions based on the use of the ICTs is confirmed, especially in occasional smokers^(^ 29 ^)^. Apart from that, these interventions are more effective if they are adapted to the smokers’ characteristics and to the quitting phase in which they are^(^ 30 ^)^, if they use applications in their mobile devices, or if they combine graphics with humorous and stimulating elements, and with challenging games^(^ 31 ^-^ 32 ^)^. Likewise, the participants’ follow-up of the intervention was lower than the expected based on the lack of commitment of the young population and on the demands during the intervention^(^ 33 ^)^.

The study presents a number of limitations: (i) Hawthorne’s bias, as the smoking habit is a highly sensitive topic, the subjects adopt a change in their behavior as a consequence of knowing they are being observed, (ii) in the analysis, it was not possible to consider whether the participants had made any comments or not on the web due to their low level of online participation, (iii) the results cannot be representative due to the use of convenience sampling, (iv) the low level of participation in the intervention can limit the generalization of the results, and (v) only the effect of a single intervention in a short period of time was investigated, which hinders the appearance of direct and immediate effects.

This study contributes to the growing area of research on tobacco use in university populations since it focuses on the use of the ICTs as a wide-scope tool to influence on the motivation to quit smoking, which constitutes the main axis of the therapy for quitting the smoking habit^(^ 34 ^)^. In future research studies, due to their wide-range and low cost^(^ 35 ^)^ the use of the social networks would be advisable as a strategy to attract the population susceptible to quitting smoking. Additionally, it is proposed to initiate research studies based on the ICTs with longer follow-up assessments, together with consultations to the health personnel which allow for advice and accompaniment in the process of quitting smoking^(^ 36 ^-^ 37 ^)^.

## Conclusion

Most of the Nursing and Physiotherapy students have low levels of motivation to quit smoking and of physical dependence to nicotine. The level of motivation to quit smoking does not change after performing an intervention based on the use of the ICTs
